# Supplementary material for: Patterns of Intron Gain and Loss in Fungi
Source: PLoS Biol. 2004 Nov 30;2(12):e422. doi: 10.1371/journal.pbio.0020422 (PMC532390; doi:10.1371/journal.pbio.0020422)
Supplement: Table S1 — Also available at http://genes.mit.edu/NielsenEtAl/. (4.3 MB ZIP). [file pbio.0020422.st001.zip › NielsenEtAl/html/356.html]

AN3123.1.NCU04512.1.MG00105.1.FG04030.1


```
 CLUSTAL W (1.82) Multiple Sequence Alignments - Introns Inserted


Sequence 1: NCU04512.1	2191 aa
Sequence 2: MG00105.1	1986 aa
Sequence 3: FG04030.1	2020 aa
Sequence 4: AN3123.1	2061 aa
Alignment Length: 2361 aa
Number Identitical Residues: 389 aa
Alignment Score (without introns) 26584


MG00105.1 	-~------------M0VDEAL-------~-----KLYQ~NALKLHAQGPKSYDDASKAYD
NCU04512.1	M0PTFQAINVEPEEN~VDEEIDTT----~-----KQIQ0NALKLHAQGPRFFDEASDAYN
FG04030.1 	M0PAFQAINLEPEDN~IDEQIDTTKEIH0VDEALKRFQ~HALKLHAQGPRSREAAEAAYN
AN3123.1  	M~STWVALNIEPDEA~IEEEVDDTKEIQ~IEEALKLYQ~NALKLHSQGPRYYKQAGEAYE
          	  .:  : . ....  ::* :. :..    ..: *  * :*****:***:  . *  **:

MG00105.1 	QLFESAIFKLPEAITEYERAQRPQTP---FLSQETAFSAA-LDVANPDGDPSATSLPQVY
NCU04512.1	ALFESEIFRYPESKTEYERTEERPDG---TLVVEPTLAQG-LEVGAADVDGIASTLPQTL
FG04030.1 	ELFESEIFKYREAKTDYERAERYADGQPEVSNLDPSLTDGGLDVDAGGADGVAASLAQAL
AN3123.1  	ALLDSEIFKYPESLSDHKRAALQDAEP-QVGGTTNDAVVGEAALDFNINDTTSSTLQQTI
          	 *::* **:  *: ::::*:        .          .   :     *  :::* *. 

MG00105.1 	YLAYKNHGHFRLDRIRHEARVASREGRPNIFDQDETQTEALGVLSDFEAALDRDPSDAEL
NCU04512.1	YLSFKNHGQFIVDRIKHKAR-ASKLGSDFDYDDAAIQHDAKKALDEFSAALDYDPSDADL
FG04030.1 	YLSYKNYGQFFVDKFKDASTSDTAWKEK---AQLKYHDDGRKVLDSWMTALDQDPSDPEL
AN3123.1  	YLSYKNHGQYVLDALRASLQELSKLSEDSSHLSSKIAESSTTALAQFAEALERDDTDLNL
          	**::**:*:: :* ::      :         .     ..  .* .:  **: * :* :*

MG00105.1 	WRRAARVSAFLKSARVSRYCLEAAIELDDDPAVGDVEPPSLAEGYAGEQLKNQLQVLGDD
NCU04512.1	WRKTARIAAFLKSARISRYSLEAAVELDDDPAVTDFEPPSLAEGFAGEQLKRQLEVLDDK
FG04030.1 	WRKAARFAGAINSNRIKRYCLEAAIELDDDPAVMELDPPSLAEGMAGEQLKDQLKLLGDD
AN3123.1  	WRQSARLCSALQSYRLTRFCLESVLADDDNRLEVRSEQLGLEETFAEEGLRKTLHSVQDR
          	**::**... ::* *:.*:.**:.:  **:      :  .* *  * * *:  *. : * 

MG00105.1 	MALSHPAMGPFVKYEVPDFLKRFLDPLPFLPNPIKEIAIPKSTKDEESLSRLILVAQVAD
NCU04512.1	MALSHPIMKSFREKGLPKFLERYLDPIPSLPDYAKTIAAPRPDSDEVGQPRLVMNIPSYS
FG04030.1 	MALSHPIMAPWLKKKMPALLKRHLDPIPFLPDPTKSLIPPKVSDTQAQTQEDTEMGDADN
AN3123.1  	LSVSLVPIKKP-KKALLKFFKQQSDPYPYLPSLPDNLQDLDSSRNPLAFRASTHEIKIDS
          	:::*   :    :  :  ::::  ** * **.  . :                      .

MG00105.1 	WTNVGL--------------------AIIAAFLNHGASGRGVFIEVPEVLEDEQDVVMDV
NCU04512.1	WTELGI--------------------ALVHFVAEHGFSGQAVAIQLPEGIVEDEDVQMEI
FG04030.1 	ASLTEP--------------------KSVSSWAELGAKLINCLETTAEAFDTCDGIVESS
AN3123.1  	LTWAAIGQAILTFLDDKNGTPSLAPGTSITISLPADSPELKTASITAQRRPSKAQVDENN
          	 :    ..:  :  .....:.: :..  :      .          .:       :  . 

MG00105.1 	DSELQAQIEAQVLEESRSGSAGSEKPAAATSDTQQTAANTDSTPATKPAADTTS-PGEGR
NCU04512.1	DKQLQPPEESTQEEEAAVVAEVTSAEKSGAQDTEEAESIVRETKVT--IVEEAR-AAQPT
FG04030.1 	LEDVPIETPPNEIESVASEIEVAVTKPTETEKSPEKDSTPKEQKAGSEGTEDSQKTADQS
AN3123.1  	NQDVQMDDAQSVGARSVTGAQGRELAMEHGDDQSSVDQRAEKQLIESLEVQSIQHQQSTD
          	 .::                          ..  .      .    .  .:      .  

MG00105.1 	KDSTASLG----------FRKRSQSEAGLADGGDEEAVDSKRPKR---ARRRDT-AVEEV
NCU04512.1	KDRTSSLP----------TRKRSQSAAGIPDPGDEEKGETKRSKR---TRRRET-APEEA
FG04030.1 	QKDSNGAS----------ARKRSQSAAGLPDGAEEENAMEKRSKR---VRRRETLQAEET
AN3123.1  	PQEDIKAEEDDLKYPENTSRKRSSGSAITEDQAERLRVKSRRTRLRDSLAEASTHTDDVV
          	 .      ... . ...: ****.. *   * .:.     :*.:  .:  . .*   : .

MG00105.1 	VDPSTIIANQLRPYQTADHELFQLIQETTKTLGINDPDASKKISEILESCSSEDRLSKLS
NCU04512.1	MDSATLLATQLQPFQAADQNLFQMTKNFLENLGVTDRDTLDRITEILDSCASDDRTSKIQ
FG04030.1 	TDPNTLIANQLQPYQGADQNLFQMTKSVLEHLGVEDKSTFAFITELIDSCATENRPGKIT
AN3123.1  	FDQAKYYEDLLEPYIQTDEGVFGTVGALLSKLGVEDLGTFEELRRSVASAGERKDSPVTP
          	 *  .     *.*:  :*. :*       . **: * .:   : . : *..  .      

MG00105.1 	S------QVAQDLAATISTFTMEQSKVLLNKEPAP---------TLGLNAFLEHTKPGTS
NCU04512.1	N------PATTDLREAILHFDEETASILLDGRESP---------PLSLSAFLEHSKSPSQ
FG04030.1 	N------LAACDLSSVIVNFREEVAKVFLHKNEQA---------SLGPSSFLEHAKTGSQ
AN3123.1  	VNIDNAEVLLQDLRNVLTQWDERLYQVMQQSDSLAGLQDIKSMGRSGLAVFLEHSKKTTH
          	 . ..:.    **  .:  :  .  .:: .    .. .. .: .  .   ****:*  : 

MG00105.1 	PASEKPPANE~TAGLKAFIARINGG--WFTLEDCAFRWLSALC-----------------
NCU04512.1	RAIEPPQFDD0---------------------DVVYEYVKSLA-----------------
FG04030.1 	DQPSMPSFNE~KQGLRSFARKIGGCRSWMTAEDVAYEWVRAVS-----------------
AN3123.1  	KLKLKQTFSG~EDELFDFIRAVNGS--RLHLHDVVFEWLGCLLRPDYKNFLTHDNQFNDW
          	        .             ..        * .:.:: .:  .. ..  : ... .. 

MG00105.1 	----HSYTASRWSEERKRAVVHVITRLDDGIYQRLQYESQNHQDSNPSVVENQPT-----
NCU04512.1	----ESYSTEKWSDQMKVAIVQVISRLDEGIYDRVVYDLEQWQLSDASGHQPSPD-----
FG04030.1 	----QSYATAKWSDKMKLSVVQMLNKVDSTLYRKITEEMELAAGS----AERLAD-----
AN3123.1  	SLVESSYVAYQWPTTLKDVVLQLLSLEDEYIHGKLEEGMQTLEHHILEAQSGTPFRYTAK
          	:  . ** : :*.   *  :::::.  *. :: ::    :       .  .  .   ::.

MG00105.1 	---MEALAQMLFELHLDIYERVTNPNSLVDAEQRKESRTRLMRWLFLRSQLFD-----SG
NCU04512.1	---IVDLVYMLFELHLDVYERITNPNSVVEQITRTKARIRLGRWWDLATQVSR-----RR
FG04030.1 	---LETIVPMLFELHVDIYELITNPSSVVDYATRMDTKYRLGRWLDIASAYLR-----LI
AN3123.1  	HFADLEMIQAIYELHLDLYAPMNAPNNETDHRTRTLQQDRLARWSMLARSALTHFIDCCP
          	  :   :   ::***:*:*  :. *.. .:   *   : ** **  :         ..  

MG00105.1 	KAQPSEELTLRFCWAVVFATTLSDTVSPDHVLDSWRSLREVVEQAGTAEIFLPNNVVMPE
NCU04512.1	YFGTDDALTLRFLWAAVVFITVTEGVPREHILNCWHSLRDHLATTTVPDIVLPNNAVMPE
FG04030.1 	DRPANDPLIARFLWASVLVSSHAEQPLREHILLMWTSLRDHLADEKVPAISLPNNVVMPT
AN3123.1  	ERVNRERITIRHIWASTFHSNMGVDAQREHILLCLEDLKRLFSCLNEPVLSLANSSIMSE
          	     : :  *. ** ..  .       :*:*    .*:  .     . : *.*. :*. 

MG00105.1 	VSVKAVDREISKLTTMDFFLGLFKEDMGDPVAVISSLEPVLNPDSVAVVTDPDTPINGMA
NCU04512.1	ISVAAADREVSKLTTMEFFLGLFQEEQDNPVSVIDTLEPVLNPESVCINSQAAPNPTEDA
FG04030.1 	ISPAAADREISKLTTMDFFLGLFQDNMDDPVFVIDTLEPVLNPSTVCAPNQGTDSESGED
AN3123.1  	LSCEAIDQEISKLNSMDFFTRIFNPDSEDPVGLIETIEPIVEPSAVQFEEGSE-------
          	:*  * *:*:***.:*:**  :*: :  :** :*.::**:::*.:*              

MG00105.1 	QD-----------------------GESGEPTG-----VSVKECATQTLQDLWKFVRGSS
NCU04512.1	MD-----------------------EDT-EPAGN---IKSVKDCASQGLQDLWNFIEKSS
FG04030.1 	SDSSASSDTTGASTLDSSDDSTDDNDESSDASSNQKQKKSISDCASQSLKDLWKFLHNSS
AN3123.1  	------------------------------------------DRQSLQRREMGSFLDRGD
          	                                          :  :   ::: .*:  ..

MG00105.1 	TDLRLFLWKRLGDAYRAIKYNTMQFSCLLRSIEMIVDDFERESYLTASAETRTSILIHML
NCU04512.1	TDLRLLLWSRLGDAYGKIKYTTKQFSCFLRSIETIVHDFERKDYVNTPQAARKELFMTML
FG04030.1 	TELRLFLWSRLGDAYDAINYSTKKFSCCLKSIELIVSDLEGETYANTVPESRRLLFMRTL
AN3123.1  	ATLRLFLWRRLQDAYRKIDYPPKVVSCHLRSIETIIKELRSQAYVEEPGEHRQATLVRWL
          	: ***:** ** ***  *.* .  .** *:*** *: ::. : *       *   ::  *

MG00105.1 	RNLDDLLIHALHLALNDN-TSFDIIDDNHLGATAAALAKLSCILHVCAIYEDEVSIGLKQ
NCU04512.1	KALDDQIIQSLHLALNHN-NSFDIIDEEHIKTTSAALAKVSCLLHVAAMYEDEASVGIIQ
FG04030.1 	KSLDELLITALGSALNDN-TAFEIIDDAHIRSTSAALAKVNCLLHVASLCEDEVRIGIKP
AN3123.1  	KSLDGMLSKTVTTVLQESDKAYDCFDTEHVKSSMSAVAFLIRLLHSFVLYDDSVRVGQSP
          	: **  :  ::  .*:....::: :*  *: :: :*:* :  :**   : :*.. :*   

MG00105.1 	N---SNVGSSFRKSLLDLLHNFQARTWCLQYCVLKVGIGSNKELFTKPSSHLSQLLAAIH
NCU04512.1	P---PATG-SLAASFLNRLREMQVRTWSLQYTVLKMGINLHPDIFLTPENDLADYLAAVH
FG04030.1 	ERTGNAASKAAFQALKNKLKEMQIRAWCLQYTMFKAGINKD-NCIIGPENDLADYLAAIH
AN3123.1  	GIDLRAALAKSLENFKEKMRDMLVRCWVLLYTLLKEAIAQNQEMFDEPLEDRVYYLRAVH
          	      .       : : ::::  * * * * ::* .*  . : :  * ..    * *:*

MG00105.1 	QVLGLRKMCKASNRIFPKMMRVELLRQTEAEDWERDLGQVLYDLHGLKLGV---EVEDHG
NCU04512.1	QVLGLRKYCKSSNKIFLKMMRVELLKLKNIENWEDYLGQVLYDLHGLKLGVGIWDVQEHD
FG04030.1 	QVIGIRKFCKASNKIFLKVMRVELLKLKNIENWEDYLGQVLYDLHGLKLGAGVWEVQDHA
AN3123.1  	NSLGLRQMCKRSRNQFLKLVKSELLALDVEQDIEADICQILFDIHGVKLSLSDHLLSDHG
          	: :*:*: ** *.. * *::: ***     :: *  : *:*:*:**:**. .   :.:* 

MG00105.1 	CPPEKLEKRNAIALVEKITILADRMSMKDLLKSELKTTIEHMSQAIGQTKSTPQMIHNLR
NCU04512.1	CPPEKLEKRQALAMVEKISLLAHRMTMKDLLKSDLKNTVESMQQAIGSAKSTAQMMHNHR
FG04030.1 	CPPEKLEKRQTMQLVERVCILANRMPMKDLLKSDLKTTIDHMQQTIGQTKSTPQMYHNLR
AN3123.1  	CVPEKLDRSTAIMMIDFVMKQAKKINIKDLSKSELKNTIEKMQQSIGTTKAVPPVSYNRR
          	* ****::  :: ::: :   *.:: :*** **:**.*:: *.*:** :*:.. : :* *

MG00105.1 	NFTEYLKKPIHPLRLYQAWKGTVSIDAVTVTTSEAALAEHGWFFLLGMIAFSKFKQVDLN
NCU04512.1	NFNEYLKTPIHPLHLYKALTGNVDLDAVTINTPDSAPAKHGWFFLLGMIALTKFKGVDLN
FG04030.1 	NFTEYLKKPIHPLRLYRALIGDVSVDAVSVNTPETILATHGWFFLLGMMALTKFKGVELN
AN3123.1  	ILNAYLKTPINPSELVRAIQGVTDLPFIPVPSQTAVIANSGWYFLLGYAALTKFR---SQ
          	 :. ***.**:* .* :*  * ..:  :.: :  :  *  **:****  *::**:    :

MG00105.1 	RRQTPGATDDLRIGQASLRSQIQFTPDRWDAWLRLAECYDYELDEAVLWTADKINKDRAE
NCU04512.1	RRQTPGALDDLRIAVTFLRLQLQYTPDRWDAWFRLAECFDYELDDMVLWTADKMNKDRAD
FG04030.1 	RRQTPGATDDLRIGATFLRLQLQFTADRWDAWFRLAECFDYELDEAVLWTADKMNKERPE
AN3123.1  	KRLNPVPTTDLDEAISWFRQDLEHNTSRWESWYRLAQVWDSKVEEDITWSADKINNNRTE
          	:* .* .  **  . : :* :::....**::* ***: :* :::: : *:***:*::*.:

MG00105.1 	LLKFQRSSIHCYSLALSHSYSMSR-----ELSDNDSEALYDLYYNFGMRMYASSREPFGM
NCU04512.1	LVKFQRQSIHCYTLALSHSYASTSDPNVYALSGGDTEALYDLYHEFGMRMYASSREPFAM
FG04030.1 	LVKFQRNAIHCYTLALSHSRNLDI-------ETHDGDPLHDLYHKFAMRMYASSREPFAM
AN3123.1  	LVTWQRNAIHCYAMAVATAAKTAE------SGPETGALLADLYTDFGIRLYSSSREPLSM
          	*:.:**.:****::*:: :                   * *** .*.:*:*:*****:.*

MG00105.1 	EPFKHSEQERFFIDTGESENGTYKKILHMEMTDYQVWKYAASLFRKAMARRPKDWK2SAY
NCU04512.1	EPFMHSDQHRFFIEISG--NGTYKEILHNQMSDYQVWKFAASLFRKAMAGKPKDWK~NPY
FG04030.1 	EPFQHSDQERYFIEDMG--IGTFKRILHSQMNKYKVWKFAAKLFRMAMDRKPDNWK2NPY
AN3123.1  	AAFSVADFTRHFSNEES--QQMYEGRPFKEMKVYNVWRLAAFLLRQALVDKPKNWM2THY
          	 .*  ::  *.* :        ::   . :*. *:**: ** *:* *:  :*.:*  . *

MG00105.1 	MYSKCLWKMYATPDDRLPESTKRTKPGKELLISALEKAVEVVSWLPKPRHGQDPVIEPHY
NCU04512.1	MIAKCLWKMYQKPEDELDEKNRHGRPTVQAVIKALEKTVEVVRTLPKPRHGQDPILEPHY
FG04030.1 	MLSKCYWKMYQTPDDQLDVKDITSKIGLMTLIDTLKKAVEVAHNARRGRN-TDPIFEPHY
AN3123.1  	MLSKCLWKMFSCDDS---VRGSSKRISLDSVLDSLLDTIDALPQRKDSRS--EPIFEPHY
          	* :** ***:   :.         :     ::.:* .:::.       *   :*::****

MG00105.1 	KILVVLYKLVSRGDMT~AQEAADVLQRQPYAVRRGEDVLIGDGDSDDKEEWEEYVIKYLC
NCU04512.1	KILSVINKLVTRGDLP~HQEAADILQRQPLAPGRGERVTLEK-----DEDWHEYMLRYMR
FG04030.1 	KIVTILHKLVIRGDVP~AKDAAEILSEQPFGLEVNPDDHFAS--FSALEDWEEYIIRNLS
AN3123.1  	KLVSIIHKLVIKEVLT0PAEASKTLVATPWARKVPACEDRNS--------WKKYILDVLK
          	*:: :: *** :  :.   :*:. *   * .          .        *.:*::  : 

MG00105.1 	HLRDKDKSNWQHRMIIR~HARILFDSDENGSDGEKSDGKEVFMGAKAAFSVLRESMFTKT
NCU04512.1	HLRDKDKSNWQHRMIIR~HARLLFDGDEIGDEEDETKKEASKEAASKAFAVLRENMFTKT
FG04030.1 	KLKEKDKANWQHRIIIR~HAKILFD------EATEVSSEDRLVNAKAAFGVLRDSMFTKT
AN3123.1  	NLKNADKANWHHRMAVR0SARIIYD------------DNKGAIAAAEAKSELTQQIFTKT
          	:*:: **:**:**: :*  *::::*             :     *  * . * :.:****

MG00105.1 	MVMNVWKCDAERPGRHHVYTEQYVRFMTRLLVAMDDRVNLEALLRRIRKKGADFYHFNEL
NCU04512.1	MVMNVWKCDAERPGRHHVYTEQYIRYMARLLAVMNDRANLEAVLRRIRKKGVDFYHFSEL
FG04030.1 	MVMNVWKCDAERPGRHHVYTEQYVRLMTKLLVIMNDRGNLEQLLRRLRKKGADFYHFTDL
AN3123.1  	MTIQVWKPEYERPGRHFVYTTRYAYFFVTLLDQLDDRANLEQLLRRVRKKQGDFINHTKL
          	*.::*** : ******.*** :*   :. **  ::** *** :***:***  ** :...*

MG00105.1 	WHYCVLAYTRLIRQAYQIPTTEEE-TFKNVSLDEFEIVAERITEWAVKLKAEEHSAFDAM
NCU04512.1	WQHGVQIYLKMIRKAFNIPANDED-PFKNVAPEDFEAVAEKIAEWVITPEAESHAALNAM
FG04030.1 	WQSCCMAYLKLLRQGYNVSPVADD-AFKALSSEEFEVVGERIADWAASDGAEN-PLFNCM
AN3123.1  	WEDMCLTYARVIRRAAGISEGHEESVFKPIGWEEFSSNTARLEELP-QLAPESPSLLELL
          	*.     * :::*:.  :.   ::: ** :. ::*.    :: :      .*. . :: :

MG00105.1 	KEAIELKKLNANLTKATPIDDLVSDCYCLIYSEVGSDLPGPPPAQILEDRAKQKE----A
NCU04512.1	KEAIELKKLNANLMKAGPIDDLINDCYSIIHHEMVPEQTASETPQGGDEHSRAGEGELPT
FG04030.1 	KEAIEFKKLNANLMKVAPIDDLLYDCYSRIYSEIAKTLPGPDPSKVVEERHHAKEVAAQL
AN3123.1  	RDAIELKKLNNNLMKVALLEDLIADVYSRIYEVNLPILLEQVDEENKEKMKVDHILMTAD
          	::***:**** ** *.  ::**: * *. *:             :  :.           

MG00105.1 	AAAASAEERPTGLNSFLSALDTK---------ANSSRAGSEAPDKTAVEQSSRP------
NCU04512.1	KPAAEGEEPKSMTLSILPALQERERAAAAAAASGSLRAPSEQPDKMSEKQLSFNGDITHR
FG04030.1 	EAAQTETKVTSSLASILNAPNGQ-----------ESITGTATPMETEKQEAAPR------
AN3123.1  	GAATADTSTPPTSAPASEAPAPRGRTKGIARRDIQKRAEAIVSRKVPPRAPATKAP-TTT
          	 .*    .  .   .   *   :  : . :    .  : :  . :   .  :  .  :  

MG00105.1 	RKAAGVRRPDILRKAEQAVLRAME-PPQAKTAAG-ASSARAGANGENGRKSRMSSAGSVG
NCU04512.1	GRKLGVRRPDVLRKAEQAVLRAAEGPPKALSGIT-RSSRKGSVSSGSGKKGQTTRDEGAD
FG04030.1 	SRKTGVRRPDVLRKAEQAVIRALEAPKPNKSRVGSVSSGKRGSRTPNQRASDAGSEEDGP
AN3123.1  	ETESATNATNSVGPKPVVEISVRQPPAAVVDSAGQQSDIPNSLHDSADDESELSEIDEDK
          	    ... .: :     . : . : *        . *.   .        .         

MG00105.1 	GGKTSRMSPGEAEGGGNGGDAEDEDIEMTEVADEDED------SRGPAIDNENDDGADAD
NCU04512.1	DDND--------ETDEDITRHDDEDITMKDADDEDNNRPRSRRSRRGQDDTQMADDEHEE
FG04030.1 	DSQIRREAGHDGDVDMKDAGDENEEDHGEEEHDEENEDEQDE-EHDEAAEDENAEEHGVE
AN3123.1  	LSKLAADPKMLFPNFGHRSRGSLEPDSELSAQASPSGDADVANENEEVGELEGDDREGED
          	 .:    .        .    . *     .   . .       ..    : :  :    :

MG00105.1 	SEGSSPRGSNQDSTDDESDLSDVPPDYEDNMPASLMFPNLRRSVES---AGPVATSDSEA
NCU04512.1	EAMSSPPGSVHDSADDESDLSDVPADYEDDIPPSLLFPNLRRSVDASAAGGAVAASATTP
FG04030.1 	EKADSETGSIEDSADDESDLSDVPEDYDEEVPPGLRFSNLGHQSEE--------------
AN3123.1  	EGEAGPDADLEEESMNDGDGDGDGDEAGEGEGENENENGNDGNVSG--------------
          	.   .  .. .:.: ::.* ..   :  :    .    .   . .               

MG00105.1 	IG---------SSDEGSG-VDEEEEDE---------------------------------
NCU04512.1	LTPGRPTIYSSSSESGSSSADEKAQSEPKRTPRKPSWHKSQATKAQHERPGSSSIDQHKA
FG04030.1 	----------------SSGEDADSESE---------------------------------
AN3123.1  	-------------NDEDDDVDMEAGEE---------------------------------
          	             .. ... * .  .*                                 

MG00105.1 	------------------------------------EAVLEEEDNEQEDEQDDEQEEDG-
NCU04512.1	TEGGSIGPMRWGLKTGRPRNGSGTSASAVVHGVPGTEDVEDAEEDEVEEEVEDEDEDEGE
FG04030.1 	---------------------------------------GDDEAEESDGEEEAEEEAEG-
AN3123.1  	----------------------------------------EEEAPGQDGEIEGNTTMEG-
          	                                        : *    : * : :   :* 

MG00105.1 	-------------------------EEDEEGDGDEEIEE---------------------
NCU04512.1	HEDAEETVGEVGETEEEGDEDVDVDEEMEDGEGEEDVEEGEEDHEDDEDVEAEEEDEEEA
FG04030.1 	-------------------------EETLGIEDTELADE---------------------
AN3123.1  	-------------------------TDAADGEDPEHVHR---------------------
          	                          :    :. *  ..                     

MG00105.1 	---------------------------EGGAEDEDQEMADDDE-----AEEEEAQEGE--
NCU04512.1	EEAEEDVRPGEHKDDVEEGTEGEEEGEEGGEEEEEEEGVEDAEDEEGDEEEEEADEEEGD
FG04030.1 	------------------------------DEDEDEEQPKRSGS----WAANDSIRAVGW
AN3123.1  	------------------------------QEQEQEQQVGGEQEGETVYDTELATEL---
          	                               *:*:::      . .     : : .    

MG00105.1 	-----------------------------
NCU04512.1	EEEGEGEEEEIGDEDTDDESEHDSSTRGN
FG04030.1 	-----------------------------
AN3123.1  	-----------------------------
          	
```
